# Long-Term Risk of Surgery Following First Diagnosis of Benign Prostatic Hyperplasia in Middle-Aged Men

**DOI:** 10.7759/cureus.20961

**Published:** 2022-01-05

**Authors:** Sirikan Rojanasarot, Benjamin Cutone, Samir Bhattacharyya, Kyle DeRouen, Larry E Miller

**Affiliations:** 1 Health Economics & Market Access, Boston Scientific Corporation, Marlborough, USA; 2 Clinical Research, Boston Scientific Corporation, Marlborough, USA; 3 Clinical Research, Miller Scientific, Johnson City, USA

**Keywords:** luts, surgery, lower urinary tract symptoms, bph, benign prostatic hyperplasia

## Abstract

Objective

Pharmacotherapy is often used to relieve lower urinary tract symptoms (LUTS) due to benign prostatic hyperplasia (BPH), yet surgery may be indicated for persistent bothersome symptoms. BPH is common among older men, yet the burden of BPH among middle-aged men may be under-recognized. This study examined the 5-year risk of BPH surgery among middle-aged men following the first BPH diagnosis.

Methods

Using the IBM MarketScan Commercial Claims and Encounters Database, males aged 35 to 64 years with a first-time primary diagnosis of BPH who were prescribed oral medication for LUTS were identified. The primary outcome was the risk of BPH surgery within five years of the first BPH diagnosis, which was analyzed using Kaplan-Meier methods. The influence of patient demographics, comorbidities, and medication use on the risk of BPH surgery was explored using a Cox proportional hazards model.

Results

Four thousand five hundred ten eligible men, 460 underwent BPH surgery within five years of BPH diagnosis. The most common surgical procedures were transurethral resection of the prostate and laser enucleation. The risk of BPH surgery over five years following BPH diagnosis was 10.2% (95% CI: 9.4% to 11.1%). In a multivariable Cox proportional hazards regression analysis, patient age was the primary factor associated with higher surgery risk. Compared to men aged 35 to 44 years, the hazard ratio for BPH surgery was 3.9 (95% CI: 1.9 to 8.4; p<0.001) among men aged 45 to 54 years, and 5.0 (95% CI: 2.4 to 10.6; p<0.001) among men aged 55 to 64 years.

Conclusions

In middle-aged men prescribed oral medication for LUTS secondary to BPH, the risk of BPH surgery was 10.2% over five years. This risk may be underappreciated and highlights the clinical need for surgical procedures with favorable risk-to-benefit profiles.

## Introduction

Benign prostatic hyperplasia (BPH) is a histological diagnosis characterized by prostatic tissue overgrowth around the urethra that affects most men during their lifetime [1]. In a systematic review characterizing the global burden of lower urinary tract symptoms (LUTS) secondary to BPH, moderate or severe LUTS were reported in 14.8% of men aged 40 to 49 years and 36.8% of men aged 70 to 79 years [2]. Medical management with an alpha-blocker or a 5-alpha reductase inhibitor is often recommended as a first-line management approach for bothersome LUTS secondary to BPH. However, the long-term clinical utility of oral medication is limited because of high discontinuation rates within the first year of initiating treatment due to intolerance or inadequate response [3-5]. For patients with bothersome LUTS who fail medical therapy, BPH surgery may be indicated. Surgical treatment options for men with BPH range from less invasive procedures with higher reintervention rates such as transurethral microwave therapy (TUMT) and transurethral needle ablation (TUNA) that can be performed in an office setting to procedures that are more invasive but with lower reintervention rates such as transurethral resection of the prostate (TURP). The risk of surgery is well characterized among older men with BPH. However, the risk among middle-aged men with bothersome LUTS remains unclear. The primary purpose of this study was to characterize the 5-year risk of BPH surgery among middle-aged men following the first BPH diagnosis. This study also explored risk factors for BPH surgery in this patient population.

## Materials and methods

Study design

In this retrospective observational study, US administrative claims from the IBM MarketScan Commercial Claims and Encounters Database were obtained on all men diagnosed with BPH between January 1, 2012, and December 31, 2018. This is the largest private-sector healthcare database in the US and includes information from employer-sponsored plans that provide health benefits to more than 15 million people annually, including employees, their spouses, and dependents. These administrative claims data were retrospectively collected and deidentified, were determined to be exempt from Institutional Review Board review, and informed consent was not required.

Patient selection

Eligible patients were males aged 35 to 64 years with a first-time primary diagnosis of BPH requiring oral medication. Patients with a BPH diagnosis were identified using International Classification of Diseases, Ninth Revision, Clinical Modification (ICD-9-CM) and International Classification of Diseases, Tenth Revision, Clinical Modification (ICD-10-CM) codes (Table 1). Incident BPH cases were defined as patients with initial BPH diagnosis in 2013, no claim with a BPH diagnosis code in 2012, and continuous coverage through December 31, 2018. Primary BPH was defined as a first-listed BPH diagnosis within the claims record; this criterion was applied to minimize the influence of unrelated confounding conditions in patients with multiple diagnoses. 

**Table 1:** Diagnostic and procedural codes used to identify BPH patients, medications, and surgery types. BPH: benign prostatic hyperplasia; HOLAP: holmium laser ablation of prostate; PUL: prostatic urethral lift; PVP: photovaporization of prostate; TUMT: transurethral microwave thermotherapy; TUNA: transurethral needle ablation; TURP: transurethral resection of prostate

CODES	CODE TYPE	
BPH DIAGNOSIS		
600.xx	ICD-9-CM	
N40.x	ICD-10-CM	
N42.83	ICD-10-CM	
BPH SURGERY		
TURP		
52601	CPT	
52630	CPT	
52450	CPT	
TUMT		
53850	CPT	
TUNA		
53852	CPT	
Laser coagulation		
52647	CPT	
PVP/HoLAP		
52648	CPT	
Simple prostatectomy		
55801	CPT	
55821	CPT	
55831	CPT	
HoLEP		
52649	CPT	
PUL		
52441	CPT	
52442	CPT	
C9739	HCPCS	
C9740	HCPCS	
MEDICATIONS		
Alpha-blockers		
alfuzosin	NDC	
doxazosin	NDC	
silodosin	NDC	
tamsulosin	NDC	
terazosin	NDC	
5-alpha reductase inhibitor		
dutasteride	NDC	
finasteride	NDC	

Outcomes

The primary outcome of this analysis was the risk of BPH surgery within five years of the first BPH diagnosis. BPH surgery included TURP, TUMT, TUNA, laser coagulation, photoselective vaporization of the prostate (PVP) / holmium laser ablation of the prostate (HOLAP), open prostatectomy, holmium laser enucleation of the prostate (HoLEP), or prostatic urethral lift (PUL) as these surgeries had Current Procedural Terminology (CPT) codes available during the study period. All CPT codes used for each surgery are listed in Table 1. Key demographic information included patient age and the geographic region at the time of diagnosis. Clinically relevant comorbidities identified within one year prior to BPH diagnosis included cardiovascular disease, hypertension, diabetes mellitus, dyslipidemia, obesity, and erectile dysfunction. A Charlson score was calculated for each patient. Oral BPH medication utilization was defined as using an alpha-blocker, 5-alpha-reductase inhibitor, or combination therapy.

Statistical analysis

Baseline patient characteristics were reported as mean and standard deviation for continuous variables and count and percentage for categorical variables. The distribution of covariates between patients who underwent BPH surgery versus those who did not were compared using the absolute average standardized difference (ASD) statistic. The ASD was calculated as the difference in means or proportions between groups divided by the pooled standard deviation. A negligible group difference was defined as an ASD of less than 0.1 [6]. The risk of BPH surgery within five years of the first BPH diagnosis was calculated using Kaplan-Meier methods. Patients free from BPH surgery were censored after five years of follow‐up. Among men who had BPH surgery within five years, their first and second procedures were reported as counts and percentages. The influence of patient demographics, comorbidities, and medication use on the risk of BPH surgery was explored using a Cox proportional hazards model. Variables that entered the univariable Cox model at p<0.10 were included in a multivariable model where variable selection used backward elimination. The hazard ratio (HR), 95% confidence interval, and corresponding P-value were reported. Two-sided P-values of less than 0.05 were considered statistically significant. Data were analyzed using Instant Health Data software (Panalgo, Boston, MA, USA) and R v3.2.1 (R Foundation for Statistical Computing, Vienna, Austria), and figures were developed with Stata v16 (StataCorp, College Station, TX, USA).

## Results

**Figure 1:**
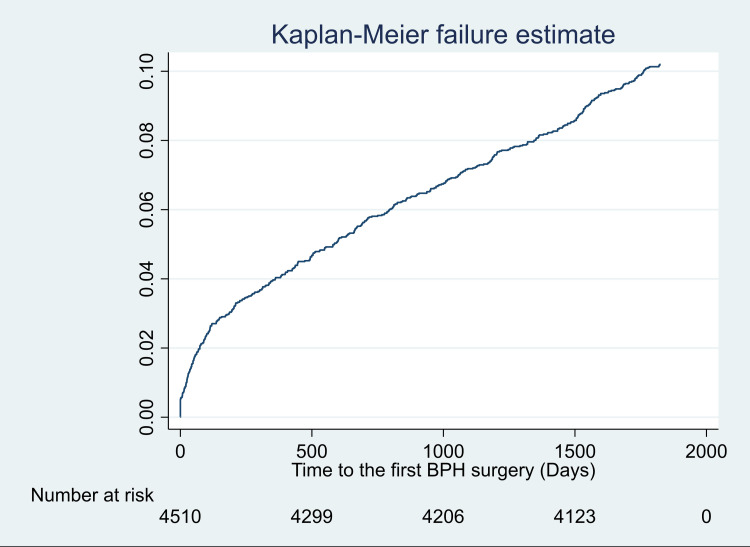
Cumulative incidence of benign prostatic hyperplasia (BPH) surgery over 5 years following first BPH diagnosis in younger males. The Kaplan-Meier risk of BPH surgery within 5 years of first BPH diagnosis was 10.2% (95% CI: 9.4% to 11.1%).

**Figure 2:**
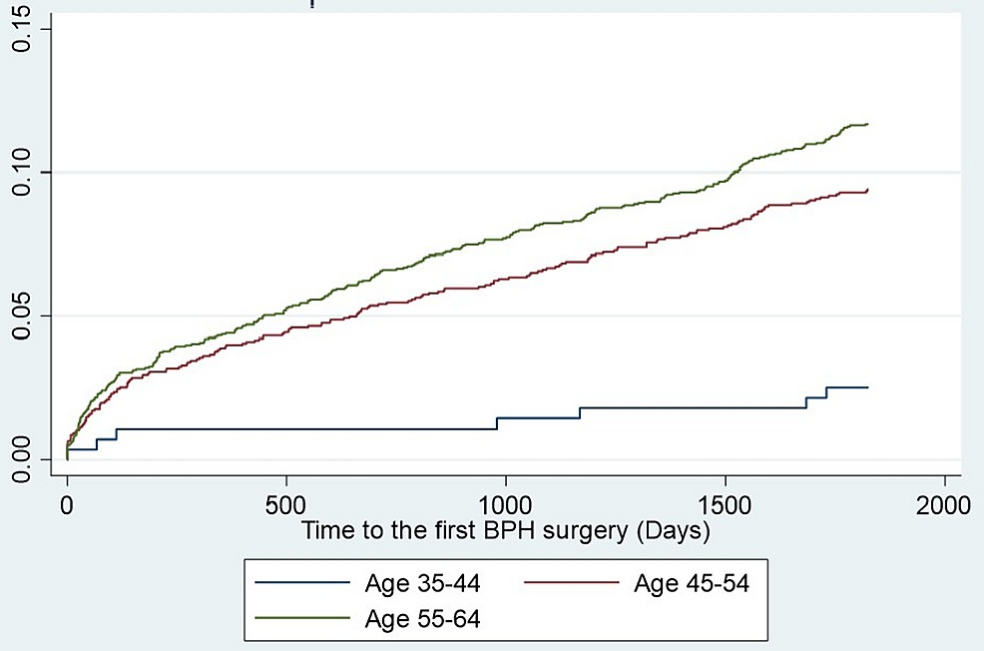
Cumulative incidence of benign prostatic hyperplasia (BPH) surgery over 5 years following first BPH diagnosis in younger males by age group. The Kaplan-Meier risk of BPH surgery within 5 years of first BPH diagnosis in men aged 35 to 44 years, 45 to 54 years, and 55 to 64 years was 2.5%, 9.4%, and 11.7%, respectively.

A total of 4,510 men met the inclusion criteria for the study (Table 2). The mean patient age was 54 years, and the most common comorbidities were dyslipidemia (39%) and hypertension (37%). Baseline patient characteristics were comparable among men who underwent BPH surgery within five years of the first diagnosis versus men who did not receive surgery (Table 3). Over five years following BPH diagnosis, 460 men underwent BPH surgery, with 47 of these men requiring a second BPH surgery. TURP and laser ablation were the most common surgical procedures (Table 4). The Kaplan-Meier risk of BPH surgery over five years following BPH diagnosis was 10.2% (95% CI: 9.4% to 11.1%) (Figure 1). In a univariable Cox proportional hazards regression analysis, patient age, geographical region of residence, absence of hypertension, and obesity were identified as potential predictors of BPH surgery and included in a multivariable model. Patient age was the strongest predictor in the multivariable model, explaining 82% of the variation (Table 5). Specifically, compared to the reference group consisting of men aged 35 to 44 years, the hazard ratio for BPH surgery was 3.9 (95% CI: 1.9 to 8.4; p<0.001) among men aged 45 to 54 years and 5.0 (95% CI: 2.4 to 10.6; p<0.001) among men aged 55 to 64 years. The corresponding Kaplan-Meier risk of surgery was 2.5% in men aged 35 to 44 years, 9.4% in men aged 45 to 54 years, and 11.7% in men aged 55 to 64 years (figure 2). Statistically significant increases in surgical risk were also identified when comparing patients residing in the West vs. the Northeast and among patients without hypertension versus those with hypertension. Yet, the strength of these associations was modest.

**Table 2:** Stepwise patient selection criteria for middle-aged men with first-time primary diagnosis of benign prostatic hyperplasia requiring oral medication.

Step	Remaining Sample	Exclusions
1	48,505,593 beneficiaries in 2013	48,029,496 with no BPH diagnosis
2	476,097 BPH cases	1,332 female cases
3	474,765 men with BPH cases	189,943 without primary BPH diagnosis
4	284,822 men with primary BPH cases	99,106 with BPH diagnosis 12-month pre-index BPH date
5	185,716 primary incident BPH cases	2,245 under 35 years of age 1,536 with BPH surgery 3,265 with malignancy
6	178,670 primary incident BPH cases absent confounding conditions	153,061 without 6 years of continuous coverage
7	25,609 cases with 6 years of continuous coverage	21,099 without BPH medication claims 12-month pre-index BPH date
8	4,510 cases included in the analysis	

**Table 3:** Baseline patient characteristics. 1: Data available for 4,502 (99.8%) patients overall, including 460 (100%) patients treated with surgery and 4,042 (99.8%) patients treated without surgery.

Characteristic	All patients (n = 4510)	Surgery (n = 460)	No surgery (n = 4050)	Standardized Difference
Age (years)				
35-44	279 (6.2%)	7 (1.5%)	272 (6.7%)	-0.22
45-54	1,827 (40.5%)	172 (37.4%)	1,655 (40.9%)	-0.12
55-64	2,404 (53.3%)	281 (61.1%)	2,123 (52.4%)	0.21
Geographic region^1^				
Midwest	1,082 (24.0%)	122 (26.5%)	960 (23.8%)	0.04
Northeast	931 (20.7%)	83 (18.0%)	848 (21.0%)	-0.04
South	1,857 (41.3%)	171 (37.2%)	1,686 (41.7%)	-0.11
West	632 (14.0%)	84 (18.3%)	548 (13.6%)	0.14
Charlson comorbidity score				
0	3,699 (82.0%)	390 (84.8%)	3,309 (81.7%)	0.07
1	460 (10.2%)	37 (8.0%)	423 (10.4%)	-0.07
2+	351 (7.8%)	33 (7.2%)	318 (7.9%)	-0.02
Cardiovascular disease	413 (9.2%)	39 (8.5%)	374 (9.2%)	0.01
Diabetes mellitus	686 (15.2%)	58 (12.6%)	628 (15.5%)	-0.10
Dyslipidemia	1,771 (39.3%)	171 (37.2%)	1,600 (39.5%)	-0.04
Erectile dysfunction	246 (5.5%)	28 (6.1%)	218 (5.4%)	0.00
Hypertension	1,688 (37.4%)	152 (33.0%)	1,536 (37.9%)	-0.10
Obesity	198 (4.4%)	13 (2.8%)	185 (4.6%)	-0.09

**Table 4:** Type of surgery within 5 years following first BPH diagnosis. BPH: benign prostatic hyperplasia; HOLAP: holmium laser ablation of prostate; PUL: prostatic urethral lift; PVP: photovaporization of prostate; TUMT: transurethral microwave thermotherapy; TUNA: transurethral needle ablation; TURP: transurethral resection of prostate.

	First Surgery (n=460)	Second Surgery (n=47)
TURP	229 (49.8%)	30 (63.8%)
PVP/HOLAP	124 (27.0%)	13 (27.7%)
TUMT	39 (8.5%)	2 (4.3%)
HoLEP	24 (5.2%)	0
TUNA	20 (4.4%)	1 (2.1%)
PUL	15 (3.3%)	0
Open prostatectomy	8 (1.7%)	1 (2.1%)
Laser coagulation	1 (0.2%)	0

**Table 5:** Predictors of surgery within 5 years following first BPH diagnosis. 1: Data available for 4,502 (99.8%) patients. BPH, benign prostatic hyperplasia.

Characteristic	Unit of Measure	HR	95% CI	P-value
UNIVARIABLE MODEL				
Age	35 to 44 years	1.0		
	45 to 54 years	3.90	1.83, 8.30	0.000
	55 to 64 years	4.89	2.31, 10.35	< 0.0001
Region ^1^	Northeast	1.0		
	South	1.04	0.80, 1.35	0.78
	Midwest	1.27	0.96, 1.68	0.09
	West	1.53	1.13, 2.08	0.006
Charlson Comorbidity Score	1 vs. 2+	0.85	0.53, 1.36	0.49
	0 vs. 2+	1.13	0.79, 1.61	0.51
Cardiovascular disease	Yes vs. no	0.91	0.66, 1.27	0.58
Diabetes mellitus	No vs. yes	1.25	0.95, 1.65	0.11
Dyslipidemia	No vs. yes	1.10	0.91, 1.32	0.34
Erectile dysfunction	Yes vs. no	1.14	0.77, 1.66	0.52
Hypertension	No vs. yes	1.22	1.00, 1.48	0.045
Obesity	No vs. yes	1.62	0.93, 2.81	0.09
MULTIVARIABLE MODEL				
Age	35 to 44 years	1.0		
	45 to 54 years	3.94	1.85, 8.40	< 0.001
	55 to 64 years	5.01	2.36, 10.61	< 0.001
Region ^1^	Northeast	1.0		
	South	1.07	0.82, 1.39	0.61
	Midwest	1.28	0.97, 1.69	0.08
	West	1.52	1.12, 2.06	0.007
Hypertension	No vs. yes	1.23	1.01, 1.49	0.042

## Discussion

In the current study, several surgical procedures were performed among men undergoing BPH surgery, with TURP being the most common procedure. BPH is a complex, heterogeneous disease such that no single treatment can be recommended for all patients. Key factors influencing treatment decision-making in BPH patients include symptom severity, comorbidities, surgical risk, socioeconomic factors, geographical location, and patient/surgeon preferences. While TURP provides clinically meaningful and durable symptom relief [7,8], it is also associated with high rates of postoperative sexual dysfunction, a significant limitation particularly among middle-aged men who may value preservation of sexual function more than older males. Ultimately, given the myriad possible patient and anatomic factors that may inform treatment decisions, a personalized approach is advocated in selecting an appropriate surgical technique in BPH patients. This research highlights the high utilization of surgery, particularly TURP, among middle-aged men who fail medical management for BPH and the need for treatments with a more favorable risk profile and reduced anesthetic risk. The American Urological Association has also echoed this sentiment [9].

The main finding from this research was that a significant number of middle-aged men were unable to sufficiently ameliorate LUTS with oral medication and resorted to surgery to attain symptom relief. Among those treated with surgery, future sexual function may be compromised with TURP as the current standard of care. Mechanical treatments utilizing stents have high failure rates and poor long-term durability and may therefore be inappropriate for use in middle-aged men [10,11]. Minimally invasive BPH treatments that leave no hardware behind may be preferable in this population given significant symptom relief, low retreatment rates, and preservation of sexual function, without the need for implantation of permanent hardware [12]. Clinical practice guidelines acknowledge the importance of patient preferences in determining the appropriate treatment for BPH. Patients prefer therapies that impact long-term disease progression over those that provide only short-term symptom improvements [13]. Development and utilization of such treatments are especially important in middle-aged men who seek more durable therapeutic options.

The conclusions of this study were strengthened by the selection of a large cohort of BPH patients treated in a real-world setting with long-term follow-up. There were also several limitations of this research. First, due to the utilization of administrative claims records, the risk of error or misclassification related to patient characteristics, diagnosis, and treatment is possible. Second, unmeasured variables may have influenced treatment selection and patient outcomes and, thus, are important sources of possible bias [14]. Examples of potentially important confounders that were unreported within the administrative claims database included medication adherence, symptom severity, sexual status, socioeconomic status, and patient/surgeon treatment preferences. Third, administrative claims were derived from employer-sponsored plans that provided health benefits from 2013 to 2018. Therefore, the analysis did not include newer BPH technologies, such as water vapor thermal therapy, robotic waterjet treatment, and other intraprostatic implants. Fourth, the results of this study were derived from patients aged 35 to 64 years and may not be generalizable to older men with BPH. Finally, a 5-year follow-up is likely inadequate to fully characterize the lifetime burden of BPH and associated treatments among middle-aged men. Future studies with follow-up periods spanning decades are recommended.

There are limitations in the current evidence guiding treatment decisions in BPH. BPH is an age-dependent disease, yet the burden among middle-aged men remains unclear. Therefore, it is important to quantify the surgical burden in this younger population and identify the variables associated with the decision to receive surgery. To the authors’ knowledge, no study has reported long-term surgery rates in this patient population. To address this limited evidence base, this study evaluated men aged 35 to 64 years who were prescribed oral medications for a first-time BPH diagnosis, which provided data on the long-term surgery rate in real-world clinical practice as well as risk factors that might influence these rates. The significant findings from this study were that: Middle-aged men have a 10% risk of a surgery over five years following their first BPH diagnosis; TURP and laser enucleation were the most common surgical procedures in this cohort, and; Old age was independently associated with a higher risk of a surgery over five years.

## Conclusions

In middle-aged men prescribed oral medication for LUTS secondary to BPH, the risk of BPH surgery was 10.2% over five years. This risk may be underappreciated and highlights the clinical need for surgical procedures with favorable risk-to-benefit profiles. Minimally invasive BPH treatments may be preferable in this population given significant symptom relief, low retreatment rates, and preservation of sexual function.
